# Meta-analysis of association between rs1447295 polymorphism and prostate cancer susceptibility

**DOI:** 10.18632/oncotarget.17627

**Published:** 2017-05-05

**Authors:** Juan Zhou, Yang Yu, Anyou Zhu, Fengchao Wang, Shuxia Kang, Yunfeng Pei, Chunping Cao, Chen Ding, Duping Wang, Li Sun, Guoping Niu

**Affiliations:** ^1^ Department of Clinical Laboratory, Affiliated to Medical College of Southeast University and Xuzhou Central Hospital, Xuzhou, People's Republic of China; ^2^ Department of Medical Oncology, Affiliated to Medical College of Southeast University and Xuzhou Central Hospital, Xuzhou, People's Republic of China; ^3^ Department of Clinical Laboratory Science, The First Affiliated Hospital of Bengbu Medical College, Anhui, People's Republic of China

**Keywords:** meta-analysis, rs1447295, prostate cancer, susceptibility

## Abstract

**Aims:**

A number of studies have found that the single nucleotide polymorphisms (SNPs) within the 8q24 region of genome were associated with the susceptibility of prostate cancer. Association between 8q24 SNP variant rs1447295 and higher risk of prostate cancer had been investigated, but those studies were incomplete and the conclusions were obscure.

**Methods:**

To better elucidate the relationship between rs1447295 polymorphism and the susceptibility of prostate cancer, we performed a more comprehensive meta-analysis about the association between rs1447295 polymorphism and prostate cancer susceptibility by collecting relevant articles published up to November, 2016 and excluding many replicated cohort data existing in previous reports, which made the conclusion more reliant and objective.

**Results:**

The results showed that there was a significant prostate cancer risk associated with rs1447295 polymorphism not only in the total groups, but also in American, European and Asian descent subgroups. Meanwhile, a comprehensive analysis about the association between rs1447295 polymorphism and prostate cancer risk were conducted by using different clinical characteristic stratifications including Gleason score, tumor stage and PSA level. The result showed that rs1447295 polymorphism was correlated with different stages of prostate cancer

**Conclusions:**

There are strong association between rs1447295 polymorphism and prostate cancer susceptibility in different ethnic groups and different prostate cancer stage, suggesting that rs1447295 might serve as a reliable biomarker for prostate cancer diagnosis.

## INTRODUCTION

Prostate cancer (PCa) is the most common cancer and the third leading cause of cancer-related deaths among males in developed countries [1]. Approximately 233,000 new cases were expected to be diagnosed with 29,480 estimated deaths in the USA in 2014 [2]. However, the underlying etiology of PCa is poorly understood. The most recognized factors associated with PCa risk include age, ethnicity, cigarette smoking, and alcohol consumption and so on. In addition, it was suggested that several genetic polymorphisms could influence an individual's susceptibility to PCa [3, 4]

Distinguishing the genetic variants that increase the risk of more advanced disease is important for improving regimens for screening, diagnosis, and the treatment of prostate cancer [5, 6]. A region on chromosome - 8q24 was first shown to confer a PCa risk in a genome-wide linkage scan of 871 Icelandic men [6, 7]. Subsequently multiple independent studies with compelling evidence demonstrated that the risk of PCa was influenced by the genetic variations in the region of 8q24 independently [6, 7].

One polymorphism in region of 8q24, rs1447295 (A/C, A was considered as risk allele), has been reported a genome-wide association studies (GWAS) association with PCa risk [7, 8]. It was reported that rs1447295 polymorphism was associated with greater tumor aggressivenessin African Americans [9] and American Whites [10], advanced stage diseases in Eastern Whites [11] and African American [9, 12]. Although there were numerous studies investigating the association between rs1447295 and the risk of PCa, the results were incomplete and cursory. Amundadottir et al. [7] showed that the rs1447295 riskallele tended to be more strongly associated with Gleason score 7–10 tumors than with Gleason score 2–6 tumors in African Americans and American Whites. However, this trend did not appear to translate to Eastern White populations [7, 13]. The relationship between PCa risk and this SNP did not appear to be affected by PSA level in Eastern Whites [11, 14]. Besides, Zheng et al. [11] showed that this allele was associated with a younger age at onset in Eastern Whites, but Schumacher et al. [15] and Zheng et al. [16] didn't show such evidence. Those contradictory results make it imperative to do a comprehensive analysis to clear up the confusion.

To meet this demand, we did a pilot study to analyze the association of rs1447295 polymorphism and different prostate cancer clinical characteristics, including Gleason score, tumor stage and PSA level. Meanwhile, we also performed a meta-analysis to offer a more comprehensive estimation of the association between rs1447295 and PCa susceptibility in three different ethic subgroups – American, Asian and European descents.

## RESULTS

### Characteristics of studies

**Figure 1:**
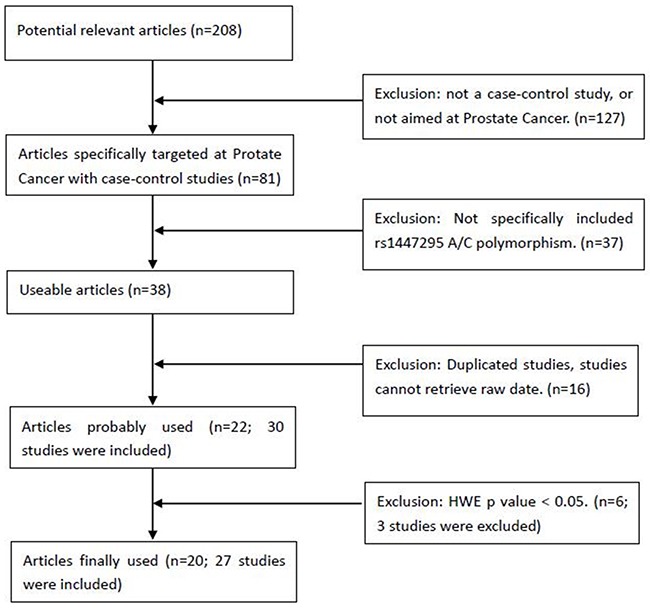
Flow chart of selecting studies with specific reasons for exclusion from the meta-analysis of rs1447295 208 articles were searched for the first-round exclusion, and 27 studies were included in the final meta-analysis for rs1447295.

A total of 208 articles were retrieved after the first search, among which 81 articles contained case-control studies targeting at prostate cancer. We removed the articles without the exact quantity information about the genotypes of rs1447295 and those studies with overlapping samples. To simplify the analysis, we restricted our study subjects only as American descent, Asian descent and European descent. Finally, 27 case-control studies from 20 articles were suitable for our meta-analysis as shown in Table 1 [Among these, 12 studies were about American descent, 7 studies were about European descent and 8 studies were about Asian descent]. All the data in these studies were related to association between 8q24 rs1447295 A/C polymorphism and human PCa susceptibility. The flow chart of selecting studies and the reasons for exclusion were presented in Figure 1. Table 1 presented the following characteristics collected from each study: year of publication and first author, race or ethnicity of samples, exact quantity of each genotype for cases and controls, HWE (Hardy-Weinberg equilibrium) p value and genotyping method. We also collected the clinical characteristic of cases, including Gleason score, tumor stage and PSA (prostate level).

**Table 1:** Characteristics of studies on association between rs1447295 polymorphism and prostate cancer included in our meta-analysis

Literature	Race/ethnic group	Case			Control			HWE p value		Genotyping method
		CC	AC	AA	CC	AC	AA	Case	Control	
2014, Cropp [29]	African-Barbadian	223	224	68	226	215	66	0.35	0.34	Infinium Human 1M-Duo
2014, Oskina [19]	Russian	291	93	8	292	50	1	0.858	0.454	Real-time PCR
2009, Meyer [25]	Caucasian German	365	107	14	370	90	2	0.079	0.157	TaqMan
2009, Beuten [18]	Caucasian	452	117	8	668	139	6	0.891	0.674	The Gold Gate Assay
2008, Salinas [17]	Caucasian American	937	288	27	994	225	14	0.382	0.752	ABI 3730xl DNA Analyzer
2008, Cheng [5]	European American	318	97	2	344	69	4	0.058	0.795	TaqMan Assay
2007, Zheng [11]	European American	1169	346	31	485	82	4	0.365	0.794	PCR
2007, Yeager(a) [8]	European American, PLCO	864	283	25	929	218	10	0.747	0.476	NA
2007, Yeager(b) [8]	European American, ACS	891	236	23	973	169	9	0.117	0.579	NA
2007, Yeager(c) [8]	European, ATBC	564	291	39	614	256	26	0.85	0.912	NA
2007, Yeager(d) [8]	European, FPCC	351	98	6	394	63	2	0.775	0.759	NA
2007, Yeager(e) [8]	European American, HPFS	469	147	9	526	106	4	0.509	0.59	NA
2007, Suuriniemi [26]	Caucasian American	435	136	11	427	107	4	0.922	0.333	TaqMan Assay
2007, Schumacher(a) [15]	Caucasian, EPIC	551	169	12	869	233	12	0.816	0.407	TaqMan Assay
2007, Schumacher(b) [15]	Caucasian, PHS	760	190	19	1054	196	14	0.084	0.156	TaqMan Assay
2007, Severi [27]	European Australian	595	212	14	586	135	11	0.322	0.319	TaqMan
2007, Haiman(a) [6]	European American	893	257	18	770	160	8	0.920	0.922	Illumina BeadStudio, Sequenom MassArray, hME or ABI TaqMan
2006, Amundadottir(a) [7]	European, Iceland	873	352	37	766	172	15	0.833	0.142	TaqMan or Centaurus platforms or by sequencing
2006, Amundadottir(b) [7]	European American	324	91	7	204	39	0	0.834	0.174	TaqMan or Centaurus platforms or by sequencing
2013, Chan [13]	Singaporean Chinese	180	92	17	94	44	5	0.260	0.957	Illumina human 1M BeadChip and Affymetrix Genome Wide Human SNP Array or PCR
2012, Joung [22]	Korean	114	67	12	127	38	3	0.611	0.936	MassArray
2011, Liu [21]	Han Chinese	514	252	38	946	378	28	0.323	0.168	MassArray
2010, Zheng [24]	Han Chinese	173	96	15	110	35	6	0.725	0.147	MassArray
2012, Liu [28]	Han Chinese	150	102	8	197	86	4	0.057	0.111	PCR and genotyped by LightScanner TMHR-I 96
2009, Liu [20]	Native Japanese	288	183	32	218	89	16	0.686	0.088	TaqMan assay
2008, Terada [9]	Native Japanese	310	172	25	254	122	11	0.856	0.421	PCR-RFLP
2007, Haiman(b) [6]	Japanese American	428	252	41	493	209	25	0.628	0.625	Illumina BeadStudio, Sequenom MassArray, hME or ABI TaqMan

### Evaluation of association between prostate cancer and rs1447295 polymorphism

**Figure 2:**
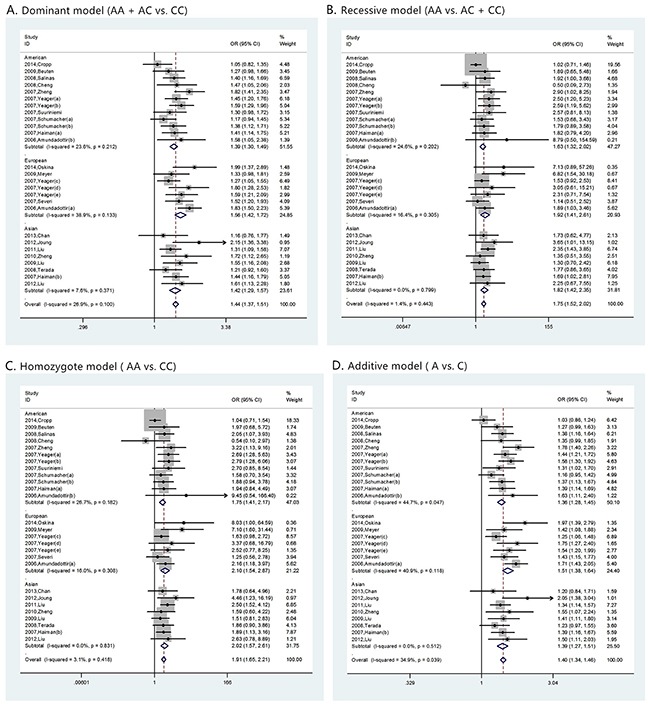
The forest plots of prostate cancer in different genetic models **(A)** dominant model (AA + AC vs. CC); **(B)** recessive model (AA vs. AC + CC); **(C)** homozygote model (AA vs. CC); and **(D)** additive model (A vs. C). For each genetic model, the analysis was performed in Asian, American and European subgroups.

Although many studies found the association between rs1447295 polymorphism and prostate cancer risk, the correlation of prostate cancer susceptibility and rs1447295 polymorphism is inconsistent. Therefore, we collected all the relevant studies with clear information of rs1447295 genotypes to address this question. Totally 27 case-control studies about association between prostate cancer and rs1447295 polymorphism were included in our analysis. For overall data, low heterogeneity was observed under all four rs1447295 gene models (for dominant model, I^2^ = 26.9%, p value = 0.100; for recessive model, I^2^ = 1.4%, p value = 0.443; for homozygote model, I^2^ = 3.1%, p value = 0.418; for additive model, I^2^ = 34.9%, p value = 0.039). Considering the low heterogeneity, M-H model was appropriate to apply. Detailed results were shown in Table 2. In general, significant prostate cancer risk was associated with rs1447295 polymorphism in all four models when all eligible studies were pooled together. For dominant model, the overall OR was 1.440 [95%CI 1.371 - 1.511; p value < 0.001]; for recessive model, the overall OR was 1.753 [95%CI 1.520 - 2.023, p value < 0.001]; for homozygote model, the overall OR was 1.913 [95%CI 1.567 - 2.606, p value < 0.001]; for additive model, the overall OR was 1.402 [95%CI 1.343 - 1.464, p value < 0.001]. The forest plots of four models were shown in Figure 2(A-D). The results indicated that there's a strong association between prostate cancer susceptibility and rs1447295 polymorphism and this was consistently observed under different genetic models.

**Table 2:** The summary results of total group, American Descent, European descent and Asian descent from dominant model (AA + AC vs. CC), recessive model (AA vs. AC + CC), homozygote model (AA vs. CC) and additive model (A vs. C) of rs1447295 A/C polymorphism

Analysis model	Pooling model	Heterogeneity		OR (95% CI)				Publication bias	
		I^2^	p value	Overall	Lower	Upper	p value	Begg's test (p value)	Egger's test (p value)
**Total Group**									
Dominant	M-H	26.9%	0.100	1.440	1.371	1.511	< 0.001	0.113	0.156
Recessive	M-H	1.4%	0.443	1.753	1.520	2.023	< 0.001	0.017	0.031
Homozygote	M-H	3.1%	0.418	1.913	1.567	2.606	< 0.001	0.016	0.033
Additive	M-H	34.9%	0.039	1.402	1.343	1.464	< 0.001	0.067	0.057
**American Descent**									
Dominant	M-H	23.6%	0.212	1.390	1.299	1.488	< 0.001	0.048	0.876
Recessive	M-H	24.6%	0.202	1.634	1.323	2.017	< 0.001	0.837	0.046
Homozygote	M-H	26.7%	0.182	1.754	1.414	2.175	< 0.001	0.784	0.057
Additive	M-H	44.7%	0.047	1.360	1.279	1.446	< 0.001	0.176	0.445
**European Descent**									
Dominant	M-H	38.9%	0.133	1.560	1.417	1.716	< 0.001	0.063	0.194
Recessive	M-H	16.4%	0.305	1.915	1.406	2.609	< 0.001	0.536	0.738
Homozygote	M-H	16%	0.308	2.105	1.543	2.871	< 0.001	0.386	0.614
Additive	M-H	40.9%	0.118	1.505	1.382	1.640	< 0.001	0.063	0.242
**Asian Descent**									
Dominant	M-H	7.6%	0.371	1.421	1.286	1.570	< 0.001	0.368	0.457
Recessive	M-H	0	0.799	1.825	1.419	2.348	< 0.001	0.072	0.042
Homozygote	M-H	0	0.831	2.021	1.567	2.606	< 0.001	0.051	0.037
Additive	M-H	0	0.512	1.386	1.273	1.510	< 0.001	0.230	0.214

Meanwhile, we performed the similar analysis for the ethic subgroup database (Table 2). For American descent subgroup which included 12 studies, the M-H model was also applied in all four models considering the low heterogeneity (for dominant model, I^2^ = 23.6%, p value = 0.212; for recessive model, I^2^ = 24.6, p value = 0.202; for homozygote model, I^2^ = 26.7 p value = 0.182; for additive model, I^2^ = 44.7%, p value = 0.047). A significant prostate cancer risk associated with rs1447295 polymorphism was also found in all four models. For dominant model, the overall OR was 1.390 [95%CI 1.299 - 1.488; p value < 0.001]; for recessive model, the overall OR was 1.634 [95%CI 1.323 -2.017, p value < 0.001]; for homozygote model, the overall OR was 1.754 [95%CI 1.414 - 2.175, p value < 0.001]; for additive model, the overall OR was 1.360 [95%CI 1.279 - 1.446, p value < 0.001]. Corresponding forest plots of four models were shown in Figure 2(A-D).

For European descent subgroup which included 7 studies, the M-H model was also applied in all four models considering the low heterogeneity. (for dominant model, I^2^ = 38.9%, p value = 0.133; for recessive model, I^2^ = 16.4%, p value = 0.305; for homozygote model, I^2^ = 16%, p value = 0.308; for additive model, I^2^ = 40.9%, p value = 0.118). A significant prostate cancer risk associated with rs1447295 polymorphism was also found in all four models. For dominant model, the overall OR was 1.560 [95%CI 1.417 - 1.716; p value < 0.001]; for recessive model, the overall OR was 1.915 [95%CI 1.406 -2.609, p value < 0.001]; for homozygote model, the overall OR was 2.105 [95%CI 1.543 - 2.871, p value < 0.001; for additive model, the overall OR was 1.505 [95%CI 1.382 - 1.640, p value < 0.001]. Corresponding forest plots of four models were shown in Figure 2(A-D). For Asian descent subgroup which included 8 studies, the M-H model was also applied in all four models considering the low heterogeneity (for dominant model, I^2^ = 7.6%, p value = 0.371; for recessive model, I^2^ =0, p value = 0.799; for homozygote model, I^2^ = 0, p value = 0.831; for additive model, I^2^ = 0, p value = 0.512). A significant prostate cancer risk associated with rs1447295 polymorphism was also found in all four models. For dominant model, the overall OR was 1.421 [95%CI 1.286 - 1.570; p value < 0.001]; for recessive model, the overall OR was 1.825 [95%CI 1.419 - 2.348, p value < 0.001]; for homozygote model, the overall OR was 2.021 [95%CI 1.567 - 2.606, p value < 0.001; for additive model, the overall OR was 1.386 [95%CI 1.273 - 1.510, p value < 0.001]. Corresponding forest plots of four models were shown in Figure 2(A-D). The result indicated that rs1447295 had strong association with prostate cancer susceptibility regardless of American, European or Asian descent.

### Evaluation of association between rs1447295 polymorphismand prostate cancer clinical characteristics

**Figure 3:**
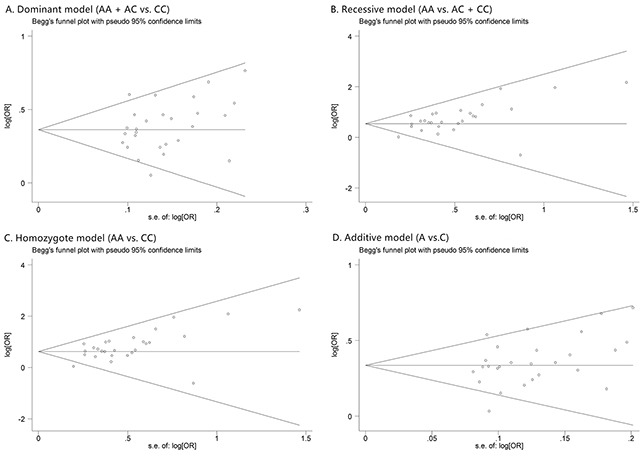
The forest plots of prostate cancer that has Gleason score <= 7 in different genetic models **(A)** dominant model (AA + AC vs. CC); **(B)** recessive model (AA vs. AC + CC); **(C)** homozygote model (AA vs. CC); and **(D)** additive model (A vs. C). For each genetic model, the analysis was performed in Asian and American subgroups.

**Figure 4:**
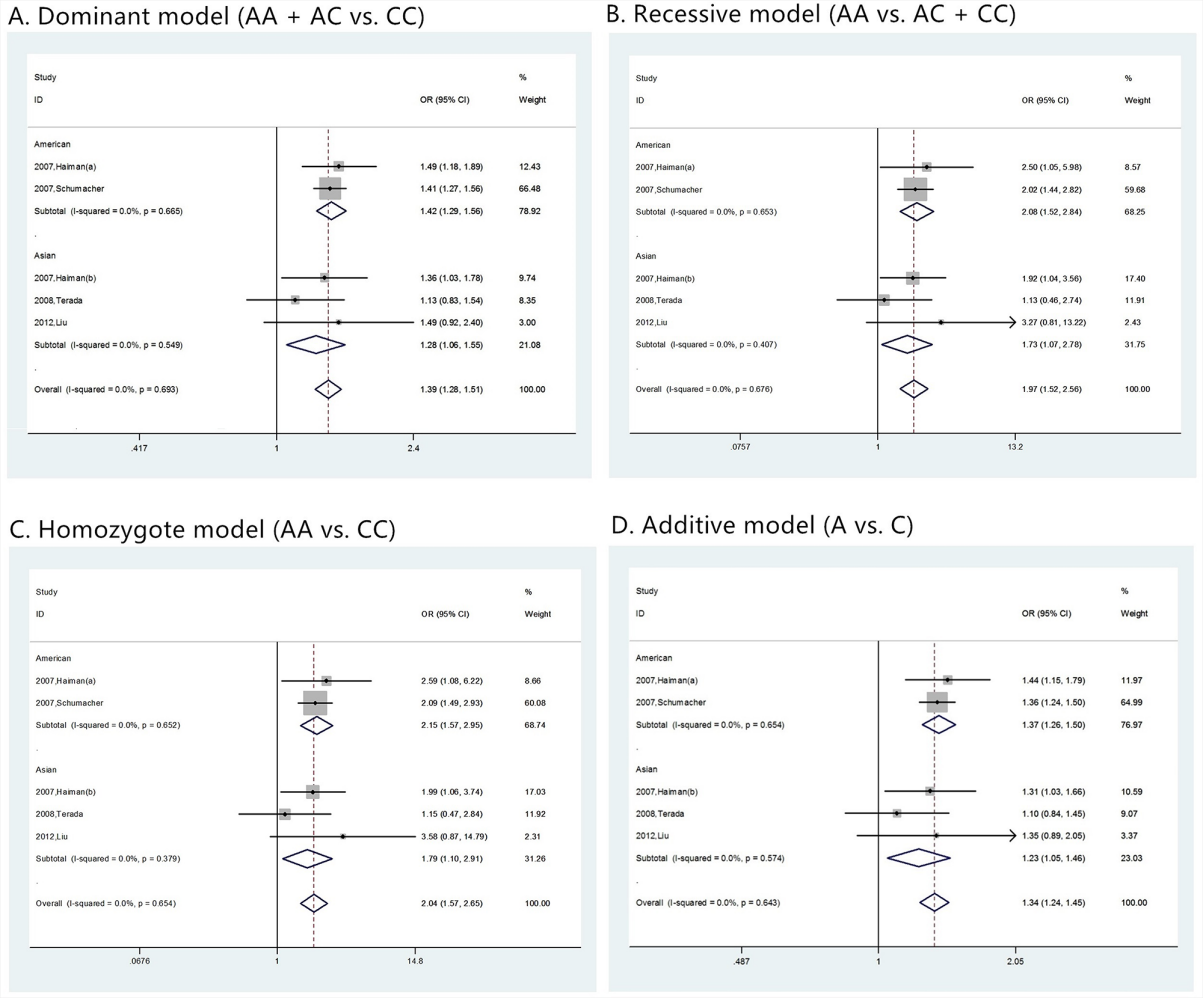
The forest plots of prostate cancer that has Gleason score > 7 in different genetic models **(A)** dominant model (AA + AC vs. CC); **(B)** recessive model (AA vs. AC + CC); **(C)** homozygote model (AA vs. CC); and **(D)** additive model (A vs. C). For each genetic model, the analysis was performed in Asian and American subgroups.

**Figure 5:**
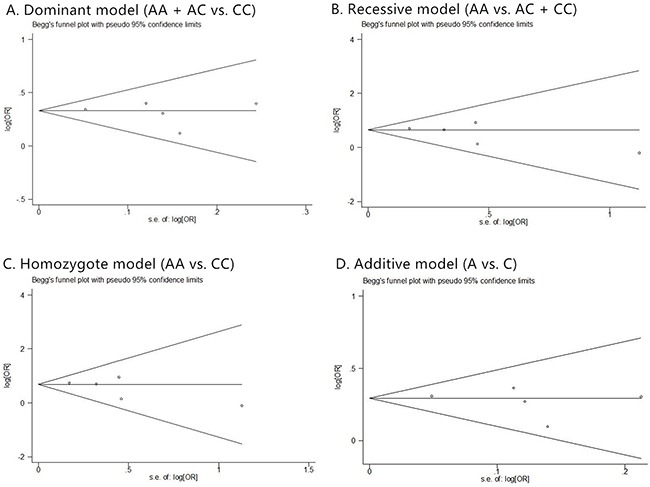
Risk evaluation of prostate cancer in different tumor stages **(A)** tumor stage 1-2, **(B)** tumor stage 3-4. The Begg's funnel plot with pseudo 95% confidence limits of prostate cancer Gleason score. **(C)** tumor stage 1-2; **(D)** tumor stage 3-4.

**Figure 6:**
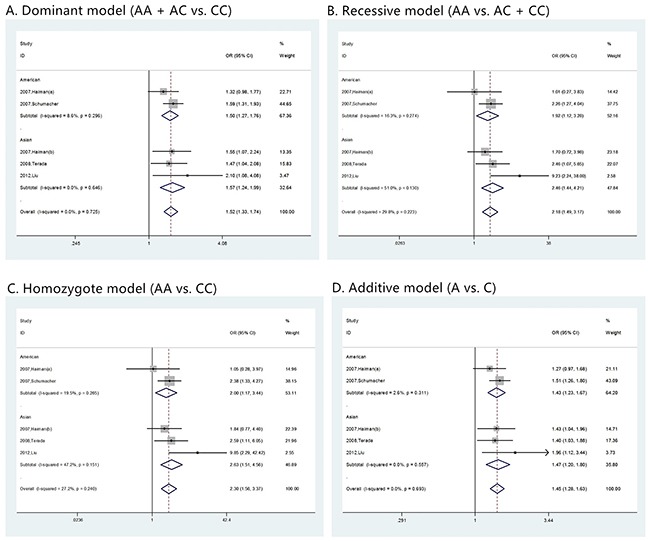
Risk evaluation of prostate cancer with different PSA levels **(A)** PSA level ≤ 10 ng/mL; **(B)**. PSA level > 10 ng/mL. The Begg's funnel plot with pseudo 95% confidence limits of prostate cancer Gleason score. **(C)** PSA level ≤ 10 ng/mL, **(D)** PSA level > 10 ng/mL.

Prostate cancer clinical characteristics, such as Gleason score, tumor stage and PSA, are important indexes for evaluating the progress of this disease. Correlation between rs1447295 polymorphismand prostate cancer clinical characteristics has been touched before but not in systematic study. Clarification of the correlation is essential to assess the diagnosis value of rs1447295 polymorphism in different prostate cancer status. Therefore, in addition to overall and subgroup analyses, we evaluated the risk of rs1447295 with different prostate cancer clinical characteristics, including Gleason score, tumor stage and PSA level, and detailed results were shown in Table 3 and 4. As for the PCa cases with Gleason score not more than 7, in dominant model, the overall OR was 1.391 [95%CI 1.279 - 1.514; p value < 0.001]; in recessive model, the overall OR was 1.968 [95%CI 1.516 - 2.555, p value < 0.001]; in homozygote model, the overall OR was 2.039 [95%CI 1.566 - 2.654, p value < 0.001]; in additive model, the overall OR was 1.341 [95%CI 1.241 - 1.449, p value < 0.001]. For cases with Gleason score more than 7, in dominant model, the overall OR was 1.520 [95%CI 1.329 - 1.738; p value < 0.001]; in recessive model, the overall OR was 2.176 [95%CI 1.491 – 3.175, p value < 0.001]; in homozygote model, the overall OR was 2.295 [95%CI 1.563 – 3.370, p value < 0.001]; in additive model, the overall OR was 1.446 [95%CI 1.281 - 1.631, p value < 0.001]. Because of the limit of the clinical characteristics data, we only did the ethic subgroup analyses in American descent and Asian descent for Gleason score (Figure 3 and 4). As for the PCa cases with tumor stage 1 to 2, the risk allelic OR was 1.287 (95% CI 1.182 – 1.402; p value < 0.001); for cases with tumor stage 3 to 4, the risk allelic OR was 1.554 (95% CI 1.362 – 1.774; p value < 0.001). As for cases with PSA level not more than 10 ng/mL, the risk allelic OR was 1.274 (95% CI 1.034 – 1.568; p value = 0.023), for cases with PSA level more than 10 ng/mL, the risk allelic OR was 1.243 (95% CI 1.019 – 1.515; p value = 0.032). Due to the limited sample size, we were not able to perform detailed ethic subgroup analyses for tumor stage and PSA level. Corresponding forest plots were shown as Figure 5 and 6. These results suggested that rs1447295 polymorphism was associated with prostate cancer risk by different clinical characteristic stratifications and might serve as a solid and sensitive marker for prostate cancer diagnosis even in the early stage.

**Table 3:** Meta-analysis of rs1447295 with Gleason score

Analysis model	Gleason score <= 7								Gleason score > 7							
	Heterogeneity		OR (95% CI)				Publication bias		Heterogeneity		OR (95% CI)				Publication bias	
	I^2^	p value	Overall	Lower	Upper	p value	Begg's test (p value)	Egger's test (p value)	I^2^	p value	Overall	Lower	Upper	p value	Begg's test (p value)	Egger's test (p value)
**Dominant**																
American	0	0.665	1.420	1.292	1.560	< 0.001	0.317	0.245	8.6%	0.296	1.497	1.271	1.761	< 0.001	0.327	0.106
Asian	0	0.549	1.285	1.064	1.551	0.009	0.602	0.756	0	0.646	1.567	1.237	1.986	< 0.001	0.296	0.460
Total	0	0.693	1.391	1.279	1.514	< 0.001	0.643	0.343	0	0.725	1.520	1.329	1.738	< 0.001	0.643	0.679
**Recessive**																
American	0	0.653	2.080	1.522	2.844	< 0.001	0.317	0.780	16.3%	0.274	1.917	1.121	3.277	0.017	0.317	0.570
Asian	0	0.407	1.728	1.074	2.782	0.024	0.602	0.831	19.0%	0.130	2.458	1.435	4.210	0.001	0.602	0.360
Total	0	0.676	1.968	1.516	2.555	< 0.001	0.355	0.393	21.8%	0.223	2.176	1.491	3.175	< 0.001	0.520	0.741
**Homozygote**																
American	0	0.652	2.151	1.571	2.947	< 0.001	0.317	0.790	19.5%	0.265	2.003	1.166	3.440	0.012	0.317	0.530
Asian	0	0.379	1.792	1.103	2.909	0.018	0.602	0.806	17.2%	0.151	2.626	1.512	4.562	0.001	0.602	0.360
Total	0	0.654	2.039	1.566	2.654	< 0.001	0.355	0.376	23.2%	0.240	2.295	1.563	3.370	< 0.001	0.520	0.739
**Additive**																
American	0	0.654	1.373	1.257	1.498	< 0.001	0.317	0.860	2.6%	0.311	1.433	1.233	1.665	< 0.001	0.317	0.139
Asian	0	0.574	1.235	1.047	1.456	0.012	0.602	0.872	0	0.557	1.468	1.198	1.799	< 0.001	0.286	0.410
Total	0	0.643	1.341	1.241	1.449	< 0.001	0.462	0.419	0	0.693	1.446	1.281	1.631	< 0.001	0.643	0.708

**Table 4:** Meta-analysis of rs1447295 with tumor stage and PSA level, *represented allelic OR comparing case to control

Study	Control		Case											
	A	C	A	C	I^2^ p value	Allelic OR* (95% CI) p value	Publication bias		A	C	I^2^ p value	Allelic OR* (95% CI) p value	Publication bias	
							Begg's test > (p value)	Egger's test (p value)					Begg's test (p value)	Egger's test (p value)
			Tumor stage 1-2						Tumor stage 3-4					
2007, Schumacher [21]	1178	9776	827	5327	0 0.474	1.287 (1.182-1.402) <0.001	0.573	0.699	237	1189	34.4% 0.206	1.554 (1.362-1.774) <0.001	0.473	0.249
2008, Terada [13]	144	630	135	513					44	120				
2013, Chan [17]	54	232	85	281					35	153				
2012, Liu [23]	94	480	37	109					20	76				
			**PSA level <= 10ng/mL**						**PSA level > 10ng/mL**					
2008, Terada [13]	144	630	84	330	18.5% 0.293	1.274 (1.034-1.568) 0.023	0.674	0.512	128	432	0 0.785	1.243 (1.019-1.515) 0.032	0.658	0.513
2013, Chan [17]	54	232	56	192					53	209				
2012, Liu [23]	94	480	47	145					40	160				

### Publication bias

**Figure 7:**
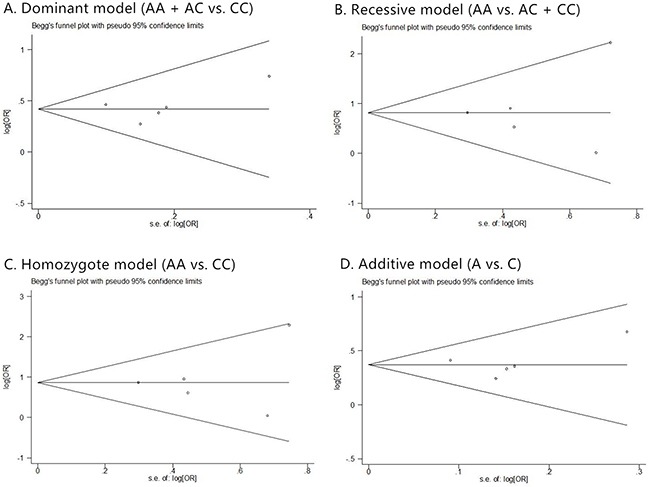
The Begg's funnel plot of prostate cancer in different genetic models **(A)** dominant model (AA + AC vs. CC); **(B)** recessive model (AA vs. AC + CC); **(C)** homozygote model (AA vs. CC); and **(D)** additive model (A vs. C).

Funnel plots were inspected to evaluate the possibility of reporting a publication bias for skewness. For the most part these did not suggest the presence of significant reporting bias (Figure 7). To test the publication bias of the literature, both Begg's test and Egger's test were performed. Results of publication bias were shown in Table 2. No publication bias was observed under any model (all p values of Egger's test and Begg's test were over 0.01).

**Figure 8:**
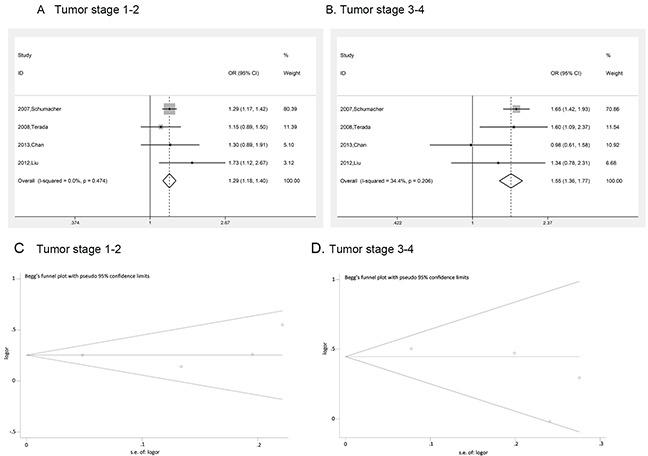
The Begg's funnel plot of prostate cancer that has Gleason score <= 7 in different genetic models **(A)** dominant model (AA + AC vs. CC); **(B)** recessive model (AA vs. AC + CC); **(C)** homozygote model (AA vs. CC); and **(D)** additive model (A vs. C).

**Figure 9:**
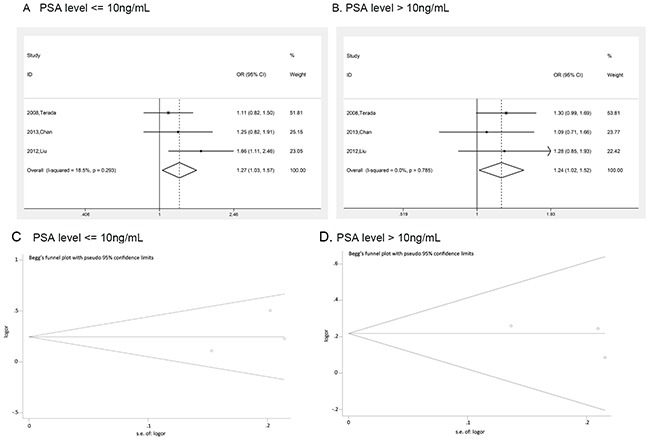
The Begg's funnel plot of prostate cancer that has Gleason score > 7 in different genetic models **(A)** dominant model (AA + AC vs. CC); **(B)** recessive model (AA vs. AC + CC); **(C)** homozygote model (AA vs. CC); and **(D)** additive model (A vs. C).

Similar evaluation was also conducted in analysis of clinical characteristics, and no publication bias was observed too. Corresponding funnel plots were shown as Figure 8, 9, 5(C, D), and 6(C, D).

## DISCUSSION

Even though there had been meta-analysis related to association between rs1447295 and prostate cancer before, we performed a more detailed analysis. We removed replicated cohort data in previous study and enlarged the data pool with the mostly recent research (2006 to 2016) in our analysis which made the conclusion more scientific and reliable. Most importantly, we found significant association between rs1447295 polymorphism and the risk of prostate cancer in total groups and also in both European descent and Asian descent. Meanwhile, our results also showed that rs1447295 polymorphism was associated with different prostate cancer clinical characteristics, providing an important evidence to use rs1447295 polymorphism as a useful marker for prostate cancer diagnosis even in the early stage.

The most recent meta-analysis for rs1447295 and PCa risk was done in 2012 [17], our analysis updated the data collected from 2006 to 2016 and focus on European descent, American descent and Asian descent. Compared with the previous meta-analysis [17], we incorporated three new articles in our analysis of Asian group. Distinct from previous analysis which only included the case and control number as the dataset but did not analyze the correlation according to rs1447295 genotypes, our study analyzed the correlation of PCa risk with rs1447295 in four different genotypes. In the four different gene models of rs1447295, the frequency of PCa significantly increased in European descent, American descent and Asian descent.

Analysis of the association between risk allele at rs1447295 and PCa clinical features showed rs1447295 polymorphism tends to correlate with advanced prostate cancer risk. For example, rs1447295 polymorphism may be associated with greater tumor aggressiveness in African Americans [9] and American Whites [10], advanced stage diseases in Eastern Whites [11] and African American [9, 12]. The rs1447295 risk allele tended to be more strongly associated with Gleason score 7–10 tumors than with Gleason score 2–6 tumors in African Americans and American Whites [7]. However, this trend was not observed in Eastern White populations [7, 13]. In addition, the correlation between rs1447295 polymorphism and PCa risk did not appear to be affected by PSA level in Eastern Whites [11, 14]. Zheng et al. [11] showed that this allele was associated with a younger age at onset in Eastern Whites, but Schumacher et al. [15] and Zheng et al. [16] didn't show such evidence. It looks like that the risk allele is only associated with a younger age at onset in African Americans populations. To clear up the confusion, we evaluated the association between rs1447295 and PCa risk for cases with different clinical characteristics including Gleason score, tumor stage and PSA (prostate specific antigen) level. Our result turned out that rs1447295 polymorphism was associated with different prostate cancer clinical characteristics in American, descent, European descent and Asian descent. The interesting results suggested that risk allele at rs1447295 might be a sensitive marker for prostate cancer diagnosis. However, a more detailed study with larger sample size and more diverse population is needed to further confirm this observation in the future.

## MATERIALS AND METHODS

### Literature search and data extraction

We searched related articles through six databases, including “PubMed”, “Science Direct”, “Karger”, “Web of Science”, “Wiley Online Library” and “Springer”. To avoid omitting related literatures, “8q24” or “rs1447295” and “prostate cancer” were set as key words. The search coverage was those published in English before November, 2016. We exclude the books and other literatures that were not related with case-control study or not aimed at prostate cancer research. Then full texts of the left articles were carefully checked and we removed articles which didn't contain the exact quantity information about the genotypes of rs1447295. Finally, we noticed there’re some studies with overlapping samples so that we picked out adequate ones, and here we restricted our study subjects as American descents, Asian descents and European descents.

All data were extracted independently by two reviewers. Preliminary evaluation was conducted based on the titles and abstracts, and then full texts of potentially relevant with our studies were obtained and re-evaluated for the inclusion. The following characteristics were collected from each study: year of publication and first author, race or ethnicity of samples, exact quantity of each genotype for cases and controls, HWE (Hardy-Weinberg equilibrium) p value and genotyping method. In addition, clinical characteristic of cases such as Gleason score, tumor stage and PSA (prostate specific antigen) level were also collected, especially those with detailed genotype.

### Statistical methods

The statistical analysis was conducted with STATA 12 (Stata Corp LP, College Station, Texas, United States). In the whole process, p value less than 0.05 was considered to be statistically significant. Hardy-Weinberg equilibrium in cases or controls was evaluated by the Chi-Square test, and p value over 0.05 was considered as significant equilibrium. HWE was also taken as a data-extraction factor and those studies with HWE p value over 0.05 were chosen for further analysis.

To get a more reasonable result, four genetic models of inheritance were adopted in our analysis: dominant model (AA + AC vs. CC; A was considered as risk allele), recessive model (AA vs. AC + CC), homozygote model (AA vs. CC) and additive model (A vs. C). In dominant model, we investigated the distribution of genotype AA and AC comparing to genotype CC; as for recessive model, the distribution of genotype AA comparing to genotype AC and CC was analyzed; in homozygote model we used CC as reference genotype and investigated the distribution of AA; as for additive model the distribution of allele A comparing to C was analyzed.

For each study, the quantities of four genotypes in cases and control groups were used as pooled data. As for pooling analysis, Mantel-Haenszel (M-H) fixed-effect model was applied to analyze datasets with insignificant heterogeneity, and DerSimonian and Laird (D-L) random-effect model was suitable for datasets with obvious heterogeneity. In our analysis, the heterogeneity among studies was evaluated using I^2^ index. The higher I^2^ was, the more significant the heterogeneity was. To be specific, when I^2^ was less than 50%, we could believe there was no significant heterogeneity among pooled data, and then M-H model is applied; for I^2^ more than 75%, an obvious heterogeneity existed and D-L model should be adopted; otherwise both models could be applied. For each analysis, M-H model was used firstly to test the heterogeneity, and then an adequate model was chosen based on the test result of I^2^ value. Pooled odds ratio (OR) and 95% confidence interval were calculated with correspondent model, and corresponding forest plot was generated to summarize the result.

As for the elaborated evaluation, factors, like Gleason score, tumor stage and PSA level, were chosen and corresponding genotypes were collected, and risk allelic OR of comparing cases with controls was calculated to see whether there were differences between cases with distinct clinical characteristics.

To evaluate publication bias, Begg's funnel plot was generated based on the analysis result and database size. The more asymmetry the funnel plot looked, the more publication bias was introduced. Meanwhile, Egger's test was also performed for further investigation. For the Egger's test, the significance level was set as p value <0.01.
